# A Radiomics Model for Preoperative Predicting Sentinel Lymph Node Metastasis in Breast Cancer Based on Dynamic Contrast-Enhanced MRI

**DOI:** 10.3389/fonc.2022.884599

**Published:** 2022-06-06

**Authors:** Mingming Ma, Yuan Jiang, Naishan Qin, Xiaodong Zhang, Yaofeng Zhang, Xiangpeng Wang, Xiaoying Wang

**Affiliations:** ^1^ Department of Radiology, Peking University First Hospital, Beijing, China; ^2^ Beijing Smart Tree Medical Technology Co., Ltd., Beijing, China

**Keywords:** breast cancer, radiomics, DCE-MRI, sentinel lymph node (SLN), metastasis

## Abstract

**Purpose:**

To develop a radiomics model based on preoperative dynamic contrast-enhanced MRI (DCE-MRI) to identify sentinel lymph node (SLN) metastasis in breast cancer (BC) patients.

**Materials and Methods:**

The MRI images and clinicopathological data of 142 female primary BC patients from January 2017 to December 2018 were included in this study. The patients were randomly divided into the training and testing cohorts at a ratio of 7:3. Four types of radiomics models were built: 1) a radiomics model based on the region of interest (ROI) of breast tumor; 2) a radiomics model based on the ROI of intra- and peri-breast tumor; 3) a radiomics model based on the ROI of axillary lymph node (ALN); 4) a radiomics model based on the ROI of ALN and breast tumor. Receiver operating characteristic (ROC) curve analysis and decision curve analysis (DCA) were used to assess the performance of the three radiomics models. The technique for order of preference by similarity to ideal solution (TOPSIS) through decision matrix analysis was used to select the best model.

**Results:**

Models 1, 2, 3, and 4 yielded AUCs of 0.977, 0.999, 0.882, and 1.000 in the training set and 0.699, 0.817, 0.906, and 0.696 in the testing set, respectively, in terms of predicting SLN metastasis. Model 3 had the highest AUC in the testing cohort, and only the difference from Model 1 was statistically significant (*p* = 0.022). DCA showed that Model 3 yielded a greater net benefit to predict SLN metastasis than the other three models in the testing cohort. The best model analyzed by TOPSIS was Model 3, and the method’s names for normalization, dimensionality reduction, feature selection, and classification are mean, principal component analysis (PCA), ANOVA, and support vector machine (SVM), respectively.

**Conclusion:**

ALN radiomics feature extraction on DCE-MRI is a potential method to evaluate SLN status in BC patients.

## Introduction

Accurate assessment of the axillary lymph node (ALN) metastasis is critical for prognosis and decisions regarding treatment modalities in breast cancer (BC). Sentinel lymph nodes (SLNs) are the first station of lymph node metastasis of BC, which can accurately predict ALN status. Therefore, SLN biopsy (SLNB) is a common procedure to assess ALN metastasis, especially in patients with clinically node-negative BC (1). Although SLNB is a surgical procedure with fewer complications than ALN dissection (ALND), it can cause shoulder dysfunction, nerve damage, arm pain/numbness, and lymphedema (2). Therefore, non-invasive methods to predict SLN metastasis are desired.

The correlations between SLN involvement and numerous variables include clinical data (age, primary tumor size, and family history) and histopathological data [lymphovascular invasion, histological grade, estrogen receptor (ER) status, progesterone receptor (PR) status, and Ki-67 proliferation index] were calculated (3–5). However, histopathological information can only be available postoperatively. Therefore, non-invasive methods are greatly needed to preoperatively evaluate SLN metastasis. Imaging techniques such as ultrasound, CT, dynamic contrast-enhanced MRI (DCE-MRI), and PET are usually used for preoperative assessment in BC detection and ALN status assessment. Among these techniques, DCE-MRI is the best tool to evaluate tumor heterogeneity by analyzing the patterns of enhancement (5). Recent studies reported that radiomics models based on MRI showed good performance in predicting SLN metastasis in BC patients (6–9). In previous studies, radiomics was a non-invasive method to quantify tumoral heterogeneity through the extraction of heterogeneity from breast MRI to identify SLN status, and all these studies used the breast tumor as the regions of interest (ROIs) (6–9). However, few radiomics studies on the prediction of SLN status included special MRI features from ROIs of ALNs.

Therefore, the purpose of this study is to develop a non-invasive radiomics model with ROIs of ALNs added from preoperative DCE-MRI to identify SLN metastasis in BC patients.

## Materials and Methods

The study was approved by the institutional ethics committee [IRB approval number: 2019(170)]. The requirement to obtain informed consent was waived because this was a retrospective study.

### Patients

A total of 142 female primary BC patients with histology confirmed from January 2017 to December 2018 at our breast disease center were enrolled. The inclusion criteria were as follows: 1) patients received SLNB within 5 days after MRI examination in our hospital; 2) patients without breast disease treatment, including surgery, chemotherapy, and radiotherapy; and 3) clinicopathological data were available. The exclusion criteria were as follows: 1) occult BC; 2) artifact on DCE-MRI; 3) patients with multifocal tumors. The clinicopathological data were collected from the patients’ medical records.

### MRI Acquisition Protocol

MRI of all patients was performed using a 3.0-T system (Signa Excite, GE Medical Systems, Chicago, IL, USA) with an 8-channel breast coil. The whole MRI protocol included T1-weighted, T2-weighted, and diffusion-weighted imaging (DWI) and DCE-MRI sequence. Gadolinium contrast agent (Gd-DTPA, Magnevist, Bayer Schering Pharma, Berlin, Germany) was administered intravenously with a flow rate of 2 ml/s at the dose of 0.1 mmol/kg of body weight using a power injector, followed by a 20-ml saline flush. One pre-contrast and eight post-contrast phase images with fat saturation were included in the DCE-MRI with the following parameters: repetition time (TR) = 4.53 ms; echo time (TE) = 1.66 ms; flip angle = 10°; field of view (FOV) = 34 cm × 34 cm; matrix = 384 × 384; slice thickness = 2.4 mm; intersection gap = 0 mm; bandwidth = 62.5 Hz; single scan time = 58 s; and single-phase scanning slices = 106. Only the third post-contrast of DCE-MRI images was collected in this study.

### Radiomics Analysis

**Figure 1:**
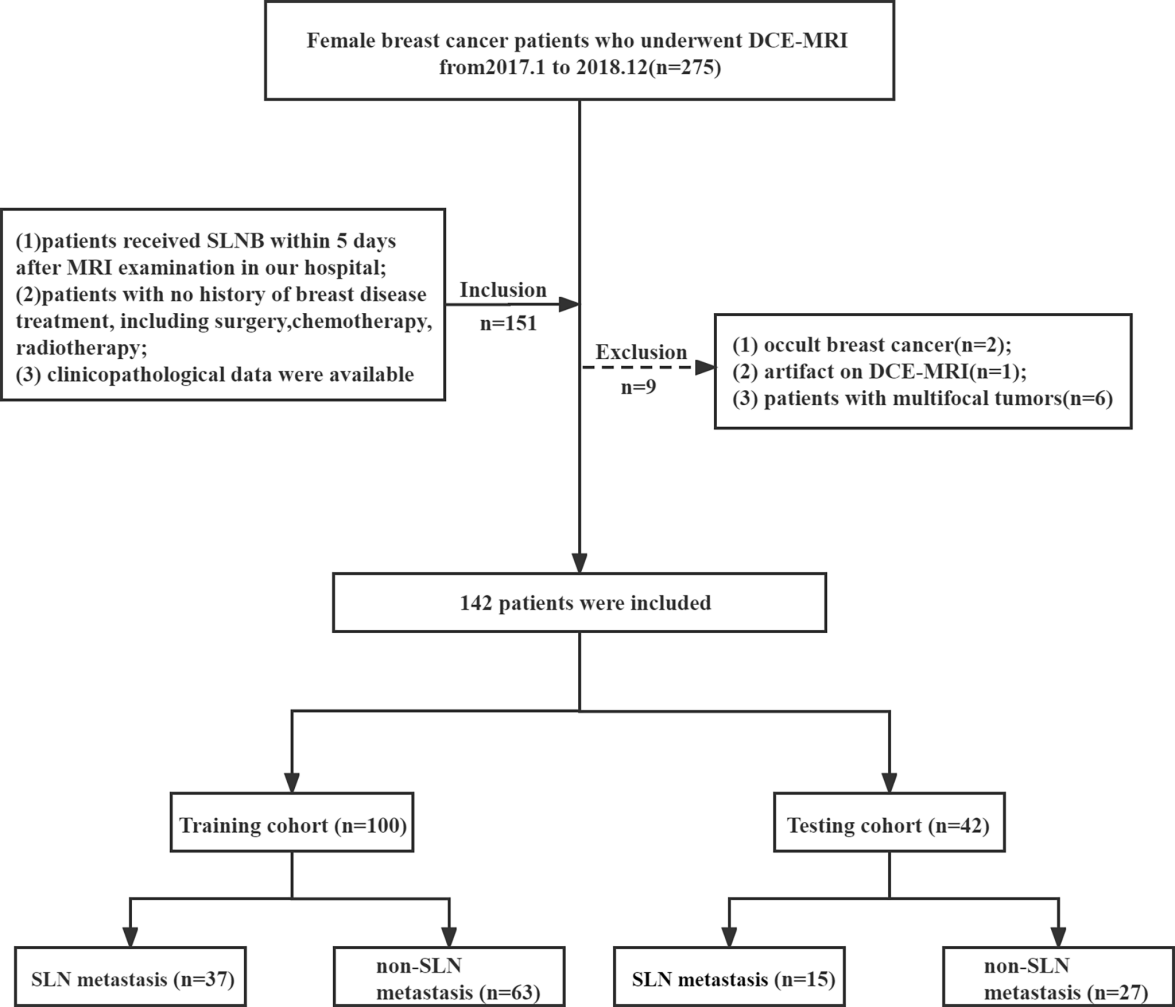
Flowchart of patient enrollment.

The patients were randomly divided into the training and testing cohorts at a ratio of 7:3. A total of 100 patients constituted the training cohort (SLN metastasis = 37 and non-SLN metastasis = 63), and 42 patients constituted the testing cohort (SLN metastasis = 15 and non-SLN metastasis = 27), as shown in **Figure 1**.

**Figure 2:**
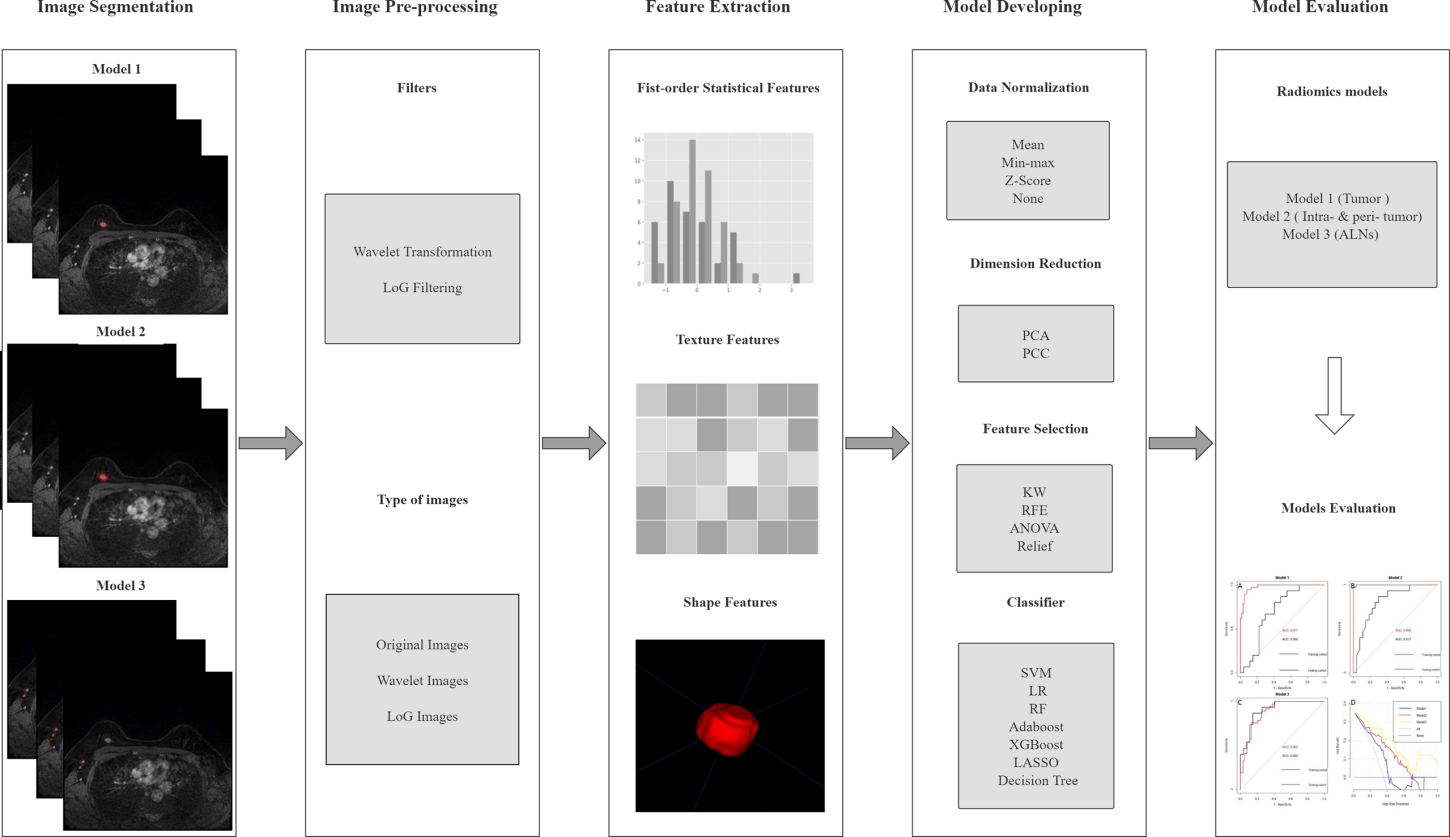
The pipeline of this study. LoG, Laplacian of Gaussian; PCA, principal component analysis; PCC, Pearson’s correlation coefficient; KW, Kruskal–Wallis; RFE, recursive feature elimination; SVM, support vector machine; LR, logistic regression; RF, random forest; LASSO, least absolute shrinkage and selection operator; ALN, axillary lymph node.

The radiomics analysis process consisted of the following steps: 1) ROI segmentation; 2) pre-processing of the acquired image; 3) feature extraction; 4) model construction. The workflow of radiomics models is summarized in **Figure 2**.

### Image Segmentation

**Figure 3:**
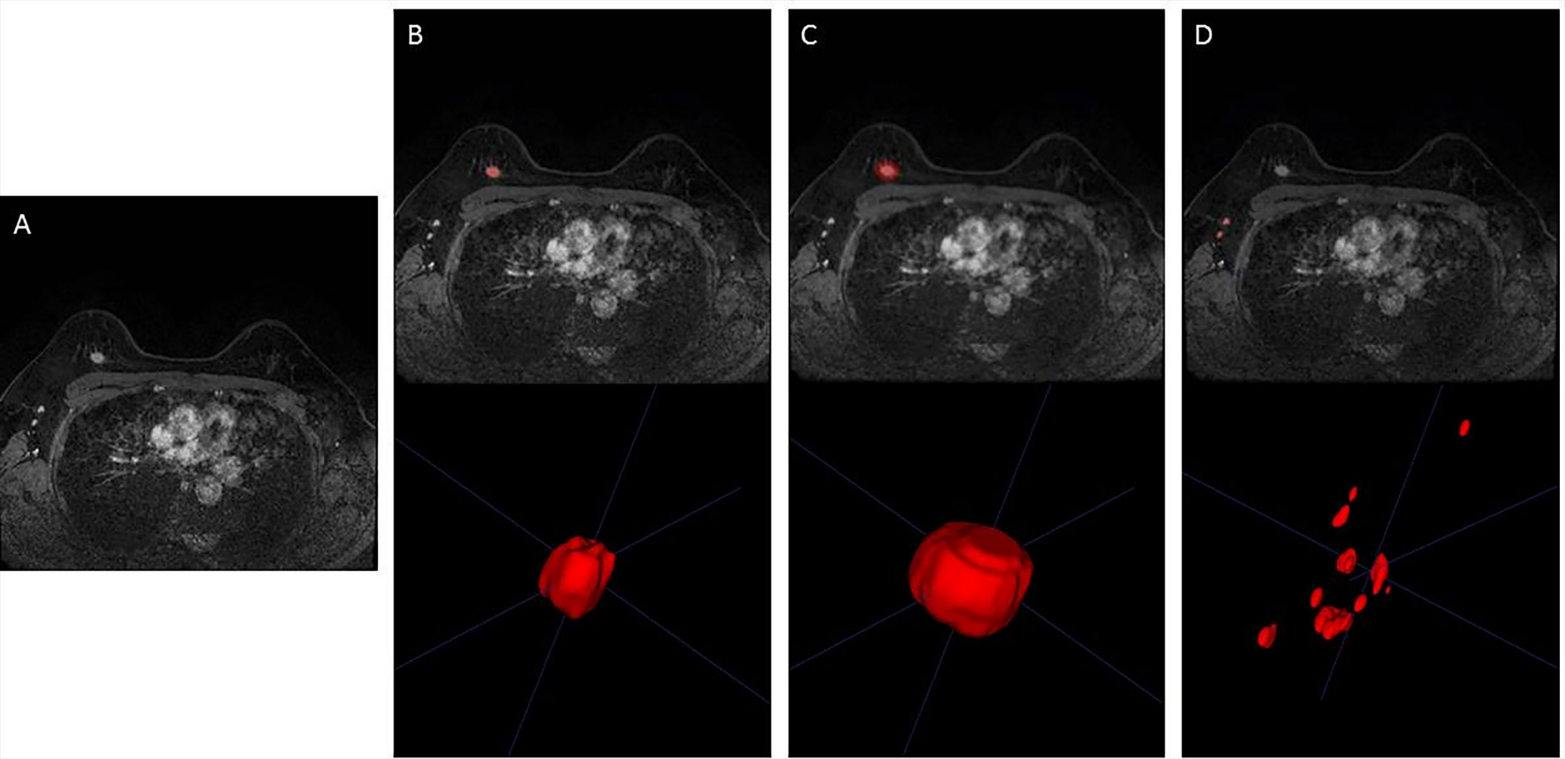
Representative image segmentation. **(A)** DCE-MRI of a 48-year-old woman with breast cancer in the third phase. **(B)** Segmentation of breast tumor (ROI of Model 1). **(C)** Segmentation of intra- and peri-breast tumor (ROI of Model 2). **(D)** Segmentation of ALN (ROI of Model 3). DCE-MRI, dynamic contrast-enhanced MRI; ROI, region of interest; ALN, axillary lymph node.

Imaging features predicting SLN metastasis were calculated based on four types of ROIs (tumor, intra- and peri-tumor, ALN, and ALN and tumor) on MRI. We used a pretrained 3-dimensional (3D) U-Net segmentation model based on deep learning in Python (v 3.6.0, https://www.python.org/) to automatically segment the breast tumor and ALNs on the third post-contrast of DCE-MRI (10). The input was the images of the third post-contrast of DCE-MRI when the tumors were most prominent, and the output was the ROIs of tumor and ipsilateral ALNs. All the automatically segmented ROIs were checked and manually modified, if necessary, by two radiologists (with more than 6 years of experience in breast MRI) based on pathological records using ITK-SNAP version 3.6.0 (www.itksnap.org). The standard range of the tumor area is the entire breast tumor, avoiding surrounding glands and blood vessels, and the standard range of the ipsilateral lymph node area is all visible lymph nodes on the affected side, excluding surrounding blood vessels. The peri-tumoral regions were obtained by dilating the ROI of the examined tumor by approximately 4 mm in 3D. The representative DCE-MRI and its corresponding ROI of the three types are shown in **Figure 3**.

### Pre-Processing and Radiomics Feature Extraction

Prior to feature extraction, all the MRI images were filtered using Laplacian of Gaussian (LoG) and wavelet algorithm. There were 3 types of images used for radiomics analysis: “original,” “LoG image,” and “wavelet image.” The pre-processing is described in detail in **Supplementary Material S1**. Then the radiomics features were extracted using the python package PyRadiomics (https://github.com/radiomics/pyradiomics). A total of 1,070 radiomics features were extracted from the ROI. The extracted features were divided into three types, including shape features (n = 14), first-order statistical features (n = 216), and texture features (n = 840) (**S2** and **Supplementary Table 1**).

### Radiomics Model Construction

The strategy used in developing radiomics models includes the following steps (**S3** and **Supplementary Table 2**): 1) data normalization (two methods: MinMax-Normalizer, Mean-Normalizer); 2) dimension reduction (two methods: Pearson’s correlation coefficient, principal component analysis); 3) feature selection (four methods: recursive feature elimination, ANOVA, Kruskal–Wallis test, and relief); and 4) classification (ten methods: least absolute shrinkage and selection operator, random forest, support vector machine, decision tree, ExtraTrees, Adaboost, logistic regression, GradientBoosting, LightGBM, and CatBoost). We used the default settings of sklearn (version 0.24.1) to train the classifier, and the specific parameters are shown in **Supplementary Material 4**. When building the radiomics models, all randomized combinations of methods were selected for use. In this way, the variable selection represents the method of choosing the most relevant radiomics features to select the most suitable model. The model with the best performance in the testing cohort was selected as the final model.

We built four radiomics models: 1) a radiomics model based on the ROI of breast tumor (Model 1); 2) a radiomics model based on the ROI of intra- and peri-breast tumor (Model 2); 3) a radiomics model based on the ROI of ALN (Model 3); and 4) a radiomics model based on the ROI of ALN and breast tumor (Model 4). For each of the four radiomics models, the model with the best performance in the testing cohort was selected as the final model. During the process of radiomics model building and testing, we use Feature Explorer Pro (FAEPro, v0.3.4) in Python (v3.6.0) (11).

### Statistical Analysis

Statistical analyses of categorical variables between the training and testing sets were carried out with the Mann–Whitney U test or chi-square test (SPSS version 23.0; SPSS, Chicago, IL, USA). Receiver operating characteristic (ROC) curve analysis was performed to assess the predictive performance of the radiomics models by calculating the area under the curve (AUC). The AUC values of the 4 models were compared by using the DeLong method. The sensitivity, specificity, and accuracy were also calculated based on the cutoff value that was maximized with the Youden index. Decision curve analysis (DCA) was used to assess the clinical practical value of the 4 models. The statistical analysis of ROC and DCA was performed by using R software (v4.1.2, www.r-project.org). The technique for order of preference by similarity to ideal solution (TOPSIS) (12) based on the performance metrics was used to reflect the balance classification and normalize the evaluation criteria (AUC, sensitivity, specificity, accuracy, geometric mean, precision, and F1 score) to select the best-performing model. For all analyses, a *p*-value <0.05 was considered statistically significant.

## Results

### Characteristics of Patients

The results of clinicopathological features are described in **Table 1**. There was no significant difference in the clinical and pathological variables between the training and test sets (*p* > 0.05).

**Table 1:** Clinicopathological characteristics of patients.

Characteristic	No. (%)			*p*-Value
	Entire set (n = 142)	Training set (n = 100)	Testing set (n = 42)	
**Age (year)^#^**	49 (44, 58)	50 (44, 54.3)	49.5 (44, 57)	0.355
**Family history of BC**				0.952
Yes	7 (5.0)	5 (5.0)	2 (4.8)	
No	135 (95.0)	95 (95)	40 (95.2)	
**Tumor location (UIQ or not)**				0.334
Yes	31 (21.8)	24 (24.0)	7 (16.7)	
No	111 (78.2)	76 (76.0)	35 (83.3)	
**Molecular subtype**				0.961
Luminal A	25 (17.6)	18 (18.0)	7 (16.7)	
Luminal B	93 (65.5)	66 (66.0)	27 (64.3)	
Triple negative	13 (9.2)	9 (9.0)	4 (9.5)	
HER2 overexpress	11 (7.7)	7 (7.0)	4 (9.5)	
**Clinical T stage**				0.938
1	5 (3.5)	3 (3.0)	2 (4.8)	
2	67 (47.2)	47 (47.0)	20 (47.6)	
3	61 (43.0)	44 (44.0)	17 (40.5)	
4	9 (6.3)	6 (6.0)	3 (7.1)	
**Histological grade**				0.566
1 (low)	74 (52.1)	52 (52.0)	22 (52.4)	
2 (intermediate)	56 (39.4)	38 (38.0)	18 (42.8)	
3 (high)	12 (8.5)	10 (10.0)	2 (4.8)	
**Histological type**				0.100
Invasive ductal carcinoma	80 (56.3)	58 (58.0)	22 (52.4)	
Invasive lobular carcinoma	42 (29.6)	25 (25.0)	17 (40.5)	
Others	20 (14.1)	17 (17.0)	3 (7.1)	

p = χ^2^ test between the training and test cohorts.

BC, breast cancer; UIQ, upper inner quadrant.

^#^Quantitative variables are expressed as median (interquartile range). The others are numbers (%) included in the dataset.

### Performance of the Radiomics Models

The top 20 features for each model were selected for modeling by feature selectors after a dimension reduction of the feature matrices. The pipelines of the three models’ development are listed in **Table 2**, and the detailed information is listed in **Supplementary Material S5**. The average inference time for each case is about 2.5 s on a personal computer with a processer of AMD PRO A10-8770 R7 (10 cores) 3.50 GHz, and RAM 16.0G.

**Table 2:** Construction process of the radiomics models.

Radiomics processes	Model 1 (tumor)	Model 2 (intra- and peri-tumor)	Model 3 (ALN)	Model 4 (ALN and tumor)
Data normalization	Mean	Mean	Mean	Mean
Dimension reduction	PCC	PCA	PCA	PCA
Features selection	Relief	Relief	ANOVA	KW
Classification	Adaboost	Adaboost	SVM	CatBoost

ALN, axillary lymph node; PCA, principal component analysis; PCC, Pearson’s correlation coefficient; KW, Kruskal–Wallis; SVM, support vector machine.

**Figure 4:**
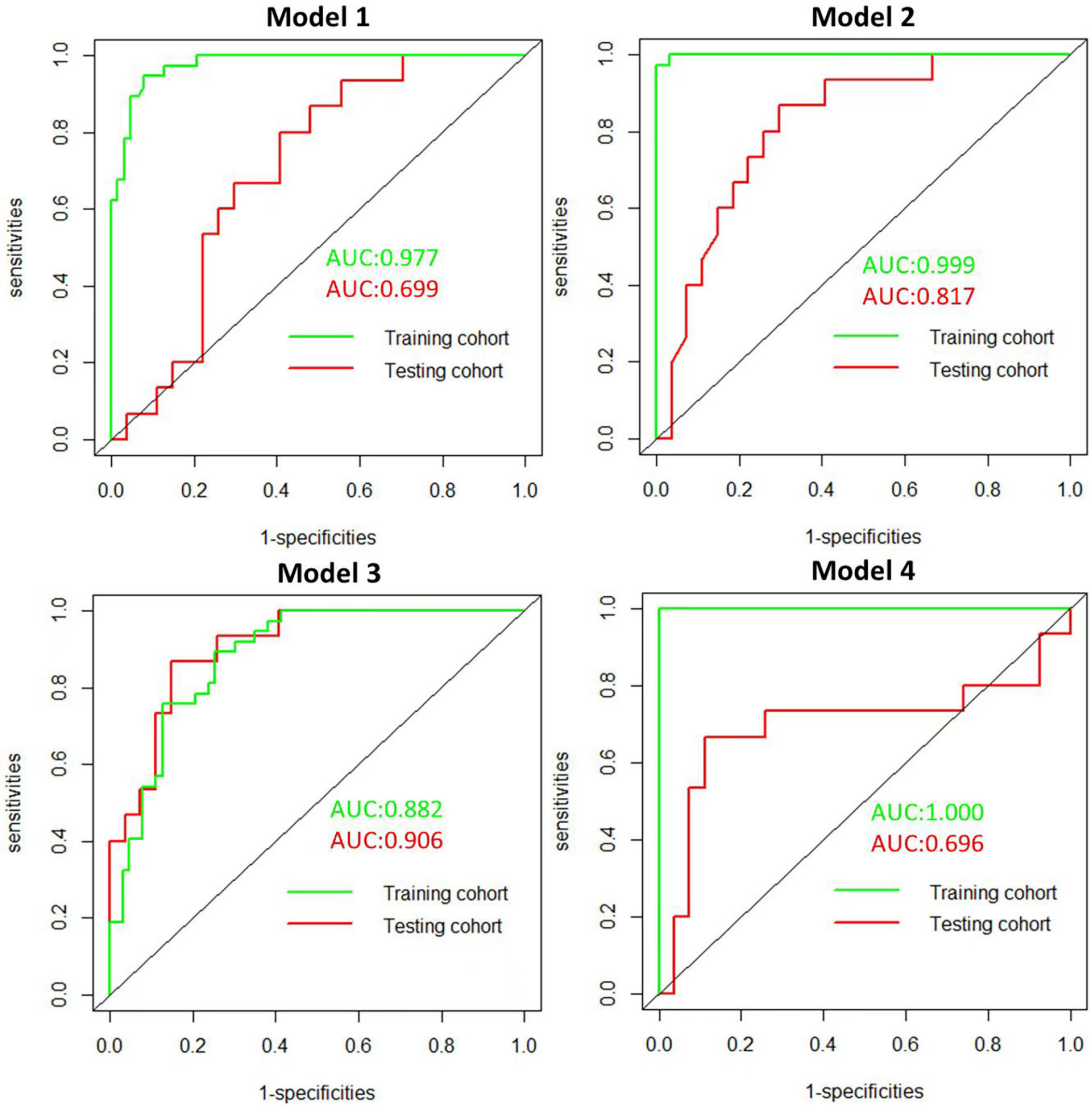
Receiver operating character (ROC). ROC of the 4 radiomics models in the training and testing cohorts.

**Figure 5:**
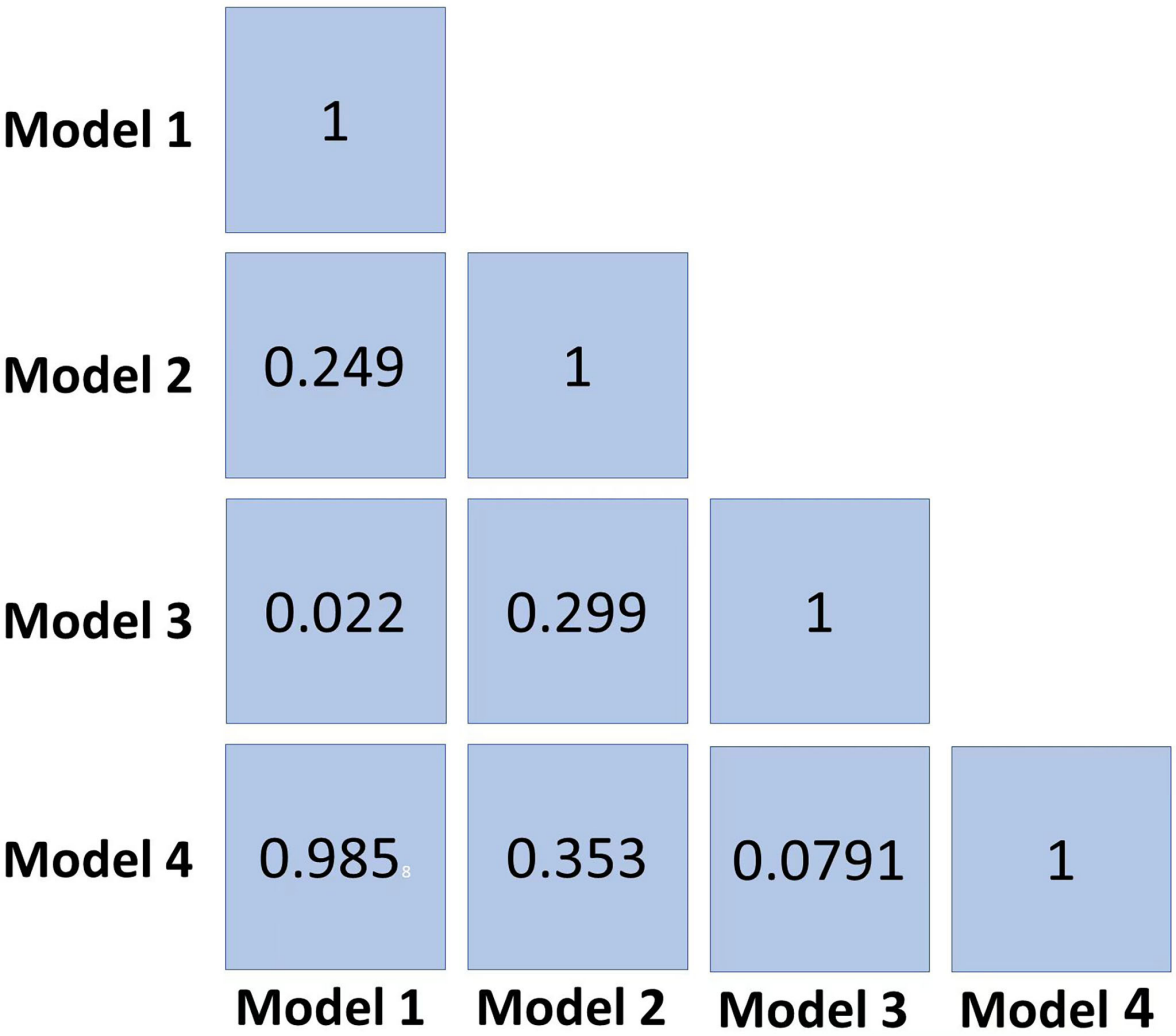
The *p*-value reflects the DeLong test between the 4 models.

**Figure 6:**
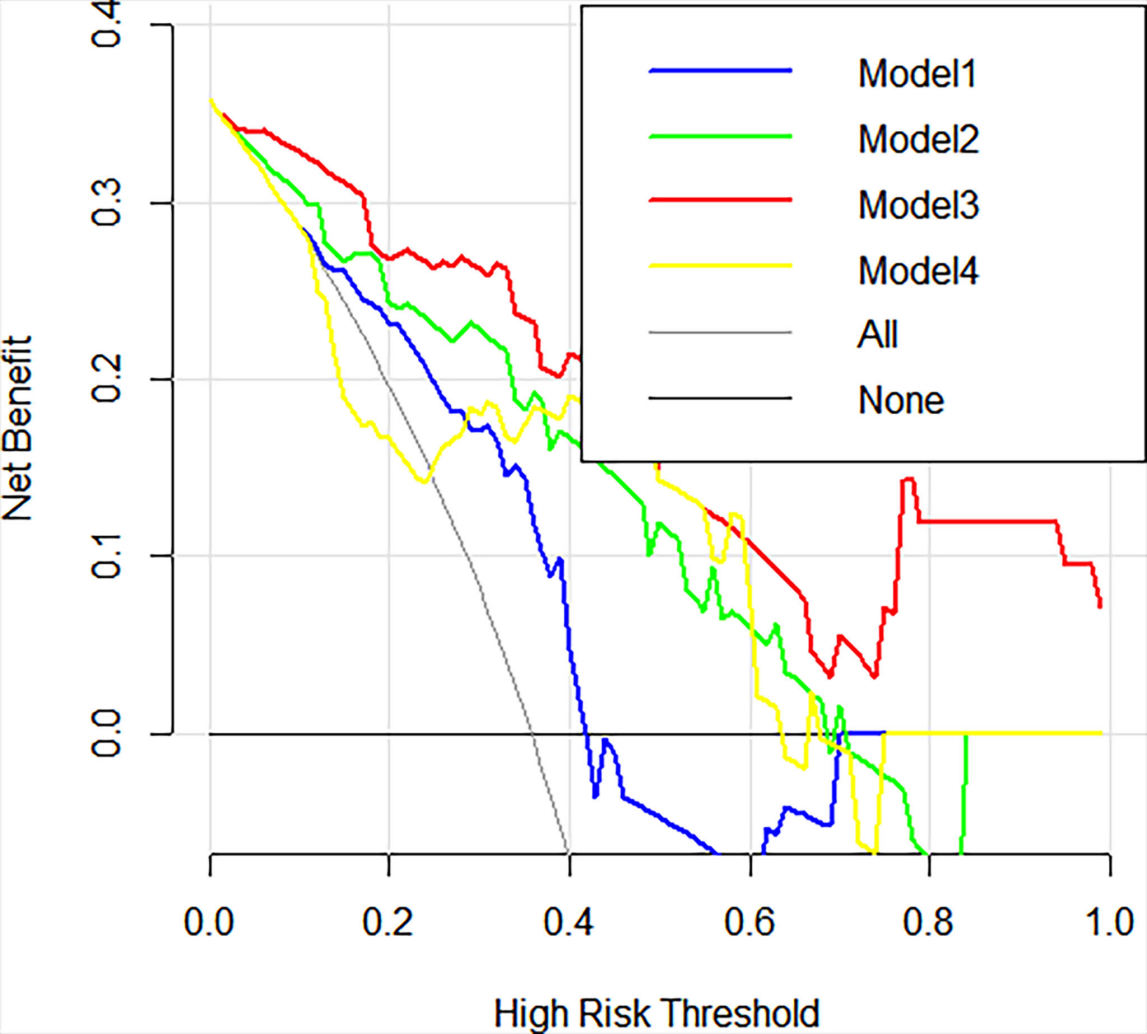
Decision curve analysis (DCA) curves. Decision curve analysis of the 4 radiomics models in the testing cohorts. The x-axis indicates the threshold probability, while the y-axis indicates the net benefit. The gray line indicates the hypothesis that all the patients achieved an SLN metastasis, and the black line indicates the hypothesis that all the patients achieved non-SLN metastasis.

The radiomics features (n = 10, 11, 6, and 6) used in Models 1, 2, 3, and 4 respectively are shown in **Supplementary Table 3**. The optimal cutoff values of 0.630, 0.537, 0.649, and 0.556 were determined by the ROC curve analysis of Models 1, 2, 3, and 4 in the training cohort. The AUC, sensitivity, specificity, accuracy, geometric mean, precision, and F1-score of the 4 models are shown in **Table 3**. The best model analyzed by TOPSIS through the decision matrix was Model 3, the 2nd was Model 2, the 3rd was Model 4, and the 4th was Model 1. Models 1, 2, 3 and 4 yielded the best performance (AUC) in predicting SLN metastasis in the testing cohort (AUC = 0.699, 0.817, 0.906, and 0.696, respectively) (**Figure 4**). Model 3 had the highest AUC in the testing cohort, and only the difference from Model 1 was statistically significant (*p* = 0.022) (**Figure 5**). In addition, the DCA showed that Model 3 yielded a greater net benefit to predict SLN metastatic stations than the other two models in the testing cohort (**Figure 6**).

**Table 3:** Performance of the 3 models in the testing cohort.

Model	AUC	Sensitivity	Specificity	Accuracy	Geometric mean	Precision	F1-score
Model 1	0.699	0.800	0.593	0.667	0.443	0.500	0.615
Model 2	0.817	0.867	0.700	0.760	0.449	0.591	0.703
Model 3	0.906	0.867	0.852	0.857	0.302	0.765	0.813
Model 4	0.696	0.667	0.889	0.810	0.289	0.769	0.714

AUC, area under the curve.

## Discussion

In this study, we designed 4 types of radiomics models to preoperatively predict SLN metastasis in BC patients. We found that the model based on the MRI features of ALN (Model 3) had the best performance in predicting SLN, which can be used as a new method for the non-invasive prediction of SLN metastasis.

To reduce the complications of ALND, including arm edema, sensory disturbances, impairment of arm mobility, and shoulder stiffness (13), SLNB is currently the standard procedure for patients with clinically node-negative BC (14). Memorial Sloan Kettering Cancer Center (MSKCC) nomogram based on clinical parameters has been the most widely used model to evaluate the SLN state (15, 16). Xiang et al. (16) validated the clinical value of the MSKCC nomogram based on cases undergoing LNB, with an AUC of 0.722 in predicting the possibility of SLN metastasis. The previous studies had proved that clinicopathological parameters, including lymphovascular invasion, the number of positive SLNs, histological grade, Ki-67 index, and ER/PR status, were independent predictors of SLN metastasis (3–5). Until data regarding the result of clinicopathological parameters after completion of surgery are available, this method of MSKCC nomogram cannot be used as a guide for SLNB.

Although previous studies had demonstrated that MRI radiomics features of the primary tumors are important biomarkers in predicting the status of SLN with BC (5–7, 17), few studies had included the radiomics features of ALNS, which had been found by Yunfang et al. (18), who observed that radiomics features extracted from ALNs could be used to predict ALN status (AUC = 0.85). The innovation of our research was that the radiomics signature included ALN besides tumor and intra- and peri-breast tumors, and the ALN radiomics signature for SLN status prediction shows the best performance in predicting SLN status with an AUC of 0.906 in the testing cohort. Many BC studies have demonstrated that biological changes in the surrounding areas of tumors can indicate important information. Ding et al. (19) found the largest improvement in AUC in the validation set when using peritumoral thicknesses of 4 mm to predict SLN metastases. In our study, the model based on intra- and peri-breast tumors (4 mm) shows a good prediction performance (AUC = 0.817), and there was no statistical difference with the model based on ALNs. Dong et al. (7) showed that the radiomics signature of tumors in the combined multiparametric MRI fat-suppressed T2-weighted imaging (FS-T2WI) and DWI can improve the performance for SLN status prediction (AUC = 0.805). However, our research was only based on DCE-MRI because it has become an important part of conventional clinical breast MRI protocol, and DWI is not available in all hospitals.

Our current study has several limitations. First, as we know, lymphatic drainage generally follows a specific path, and most of the metastatic SLNs are in axillary level I, but the boundary of this area is not clear. Therefore, the ALN radiomics feature determined by MRI is based on all visible ALN in the armpit, but due to the special position of the breast MRI examination, the axillary area may not be completely covered in the breast MRI, which may affect radiomics analysis. In addition, it is challenging to identify SLN node by node through radiological–pathological correlation in this study. Second, multifocal tumors were not included, which may be biased against patient selection. Third, only the third post-contrast of DCE-MRI images was collected, and future research will evaluate the robustness of features at multiple time points of DCE-MRI. Fourth, this study used SLNB as the gold standard for confirming SLN status, which has a certain false-negative rate. In a future study of the radiomics model, we will add 5 years of follow-up in patients with non-SLN metastases. Finally, we explored “hand-crafted” features that describe the lesion’s size, shape, texture, and enhancement patterns in this study, which may not capture the full range of information contained within the images and are limited by low reproducibility.

## Conclusion

In conclusion, this study demonstrates that ALN-based DCE-MRI signatures have the highest predictive power and clinical utility for radiomics analysis to preoperatively predict SLN status in BC patients. This non-invasive method to evaluate SLN status can guide further treatment and eliminate unnecessary invasive LN removal for those with non-SLN metastasis. However, a large amount of multicenter data and further validation on independent datasets are required to verify its predictive properties.

## Data Availability Statement

The raw data supporting the conclusions of this article will be made available by the authors, without undue reservation.

## Ethics Statement

The studies involving human participants were reviewed and approved by the Peking University First Hospital ethics committee, Institutional Review Board approval No:2019(170). This study was a retrospective study. The ethics committee waived the requirement of written informed consent for participation.

## Author Contributions

MM and YJ contributed equally to this work and share the first authorship. MM: conceptualization, methodology, validation, formal analysis, investigation, writing—original draft, and writing—review and editing. YJ: conceptualization, methodology, validation, formal analysis, investigation, and writing—review and editing. NQ: conceptualization, methodology, and writing—review and editing. XZ: methodology, formal analysis, and software. YZ: methodology, data analysis, and data curation. XPW: data analysis and visualization. XYW: conceptualization, methodology, validation, resources, writing—review and editing, and supervision. All authors listed have made a substantial, direct, and intellectual contribution to the work and approved it for publication.

## Conflict of Interest

Authors YZ and XPW were employed by Beijing Smart Tree Medical Technology Co., Ltd.

The remaining authors declare that the research was conducted in the absence of any commercial or financial relationships that could be construed as a potential conflict of interest.

## Publisher’s Note

All claims expressed in this article are solely those of the authors and do not necessarily represent those of their affiliated organizations, or those of the publisher, the editors and the reviewers. Any product that may be evaluated in this article, or claim that may be made by its manufacturer, is not guaranteed or endorsed by the publisher.
