# Supplementary material for: A Radiomics Model for Preoperative Predicting Sentinel Lymph Node Metastasis in Breast Cancer Based on Dynamic Contrast-Enhanced MRI
Source: Front Oncol. 2022 Jun 6;12:884599. doi: 10.3389/fonc.2022.884599 (PMC9207247; doi:10.3389/fonc.2022.884599)
Supplement: Supplementary file 1 [file DataSheet_1.docx]

**S1:** **Images pre-processing**

1. **Images normalization**

MR image patterns may differ between different scanners and vendors. By normalizing the image before feature calculation, this confounding effect may be reduced. However, if only one specific scanner is used, or the images reflect some absolute world value, consider disabling the normalization. Normalize Scale: 100 (This allows you to use more or less the same bin width). Bin Width: 5.

1. **First-order specific settings**

When normalizing, gray values below the mean will be negative. Shifting by 300 (3 Std Devs * 100) ensures that the majority of voxels is positive (only outliers >3 SD lower than the mean will be negative). Voxel Array Shift: 300.

1. **Image types to use**

"Original Images" indicate unfiltered images. “LoG Images”: The images filtered with the Laplacian of Gaussian filter. The LoG filter was applied to the original images to obtain fine to coarse textures (filter width: fine, σ=1.0; medium, σ=3.0; coarse, σ=5.0). So, there are 3 types of LoG images. “Wavelet Images”: The original images underwent a three-dimensional (i.e., x, y, and z directions) wavelet transformation through the *PyWavelet* package. Each image was filtered by a high band-pass filter or low band-pass in the three directions. Thus, resulting in 8 combinations of different decompositions: LLH, LHL, HLL, LHH, HHL, HLH, HHH, LLL (H means high, L means low).

**S2: Radiomics features extraction**

There were 3 types of images used in this study: Original Images (n = 1), LoG Images (n = 3) and Wavelet Images (n = 8) (See S1 for details). There were 3 types of radiomics features used in this study: (1) shape-based features (n = 14); (2) first-order statistical features (n = 18); (3) texture features (n = 24 Gray Level Co-occurrence Matrix (GLCM) +16 Gray Level Co-occurrence Matrix (GLRLM) +16 Gray Level Size Zone Matrix (GLSZM) +14 Gray Level Dependence Matrix (GLDM)) (Supplementary Table 1)[1, 2]. The shape features were only extracted from the “Original Images”, while the first-order statistical and texture features were extracted from all the three types of images. Therefore, 14 shape features, 216 ((1 Original Images + 3 LoG Images + 8 Wavelet Images) × 18) first-order statistical features and 840 ((1 Original Images + 3 LoG Images + 8 Wavelet Images) × (24+16+16+14)) texture features were used in our study[2]. We extracted all or some of these features from the region of interest (ROI) as needed.

**Supplementary Table 1. Radiomic features used in this study.**

| Feature classification |  | Features names | |
| --- | --- | --- | --- |
| Shape Features (n=14) |  | Elongation |  |
|  |  | Flatness | |
|  |  | Least Axis Length | |
|  |  | Major Axis Length | |
|  |  | Maximum 2D Diameter (Column) | |
|  |  | Maximum 2D Diameter (Row) | |
|  |  | Maximum 2D Diameter (Slice) | |
|  |  | Maximum 3D Diameter | |
|  |  | Mesh Volume | |
|  |  | Minor Axis Length | |
|  |  | Sphericity | |
|  |  | Surface Area | |
|  |  | Surface Volume Ratio | |
|  |  | Voxel Volume | |
| First-order Statistical Features (n=18) |  | 10^th^ Percentile | |
|  |  | 90^th^ Percentile | |
|  |  | Energy | |
|  |  | Entropy | |
|  |  | Interquartile Range | |
|  |  | Kurtosis | |
|  |  | Maximum | |
|  |  | Mean Absolute Deviation | |
|  |  | Mean | |
|  |  | Median | |
|  |  | Minimum | |
|  |  | Range | |
|  |  | Robust Mean Absolute Deviation | |
|  |  | Root Mean Squared | |
|  |  | Skewness | |
|  |  | Total Energy | |
|  |  | Uniformity | |
|  |  | Variance | |
| **Textural Features: Gray Level Co-occurrence Matrix (GLCM) (n=24)** |  | Autocorrelation | |
|  |  | Cluster Prominence | |
|  |  | Cluster Shade | |
|  |  | Cluster Tendency | |
|  |  | Contrast | |
|  |  | Correlation | |
|  |  | Difference Average | |
|  |  | Difference Entropy | |
|  |  | Difference Variance | |
|  |  | Inverse Difference (ID) | |
|  |  | Inverse Difference Moment (IDM) | |
|  |  | Inverse Difference Moment Normalized (IDMN) | |
|  |  | Inverse Difference Normalized (IDN) | |
|  |  | Informational Measure of Correlation (IMC) 1 | |
|  |  | Informational Measure of Correlation (IMC) 2 | |
|  |  | Inverse Variance | |
|  |  | Joint Average | |
|  |  | Joint Energy | |
|  |  | Joint Entropy | |
|  |  | Maximal Correlation Coefficient (MCC) | |
|  |  | Maximum Probability | |
|  |  | Sum Average | |
|  |  | Sum Entropy | |
|  |  | Sum of Squares | |
| **Textural Features: Gray Level Run Length Matrix (****GLRLM)** |  | Gray Level Non-Uniformity (GLN) | |
| **(n=16)** |  | Gray Level Non-Uniformity Normalized (GLNN) | |
|  |  | Gray Level Variance (GLV) | |
|  |  | High Gray Level Run Emphasis (HGLRE) | |
|  |  | Long Run Emphasis (LRE) | |
|  |  | Long Run High Gray Level Emphasis (LRHGLE) | |
|  |  | Long Run Low Gray Level Emphasis (LRLGLE) | |
|  |  | Low Gray Level Run Emphasis (LGLRE) | |
|  |  | Run Entropy (RE) | |
|  |  | Run Length Non-Uniformity (RLN) | |
|  |  | Run Length Non-Uniformity Normalized (RLNN) | |
|  |  | Run Percentage (RP) | |
|  |  | Run Variance (RV) | |
|  |  | Short Run Emphasis (SRE) | |
|  |  | Short Run High Gray Level Emphasis (SRHGLE) | |
|  |  | Short Run Low Gray Level Emphasis (SRLGLE) | |
| **Textural Features: Gray Level Size Zone Matrix (****GLSZM)** |  | Gray Level Non-Uniformity (GLN) | |
| **(n=16)** |  | Gray Level Non-Uniformity Normalized (GLNN) | |
|  |  | Gray Level Variance (GLV) | |
|  |  | High Gray Level Zone Emphasis (HGLZE) | |
|  |  | Large Area Emphasis (LAE) | |
|  |  | Large Area High Gray Level Emphasis (LAHGLE) | |
|  |  | Large Area Low Gray Level Emphasis (LALGLE) | |
|  |  | Low Gray Level Zone Emphasis (LGLZE) | |
|  |  | Size-Zone Non-Uniformity (SZN) | |
|  |  | Size-Zone Non-Uniformity Normalized (SZNN) | |
|  |  | Small Area Emphasis (SAE) | |
|  |  | Small Area High Gray Level Emphasis (SAHGLE) | |
|  |  | Small Area Low Gray Level Emphasis (SALGLE) | |
|  |  | Zone Entropy (ZE) | |
|  |  | Zone Percentage (ZP) | |
|  |  | Zone Variance (ZV) | |
| **Textural Features: Gray Level Dependence Matrix (****GLDM)** (n=14) |  | Dependence Entropy (DE) | |
|  |  | Dependence Non-Uniformity (DN) | |
|  |  | Dependence Non-Uniformity Normalized (DNN) | |
|  |  | Dependence Variance (DV) | |
|  |  | Gray Level Non-Uniformity (GLN) | |
|  |  | Gray Level Variance (GLV) | |
|  |  | High Gray Level Emphasis (HGLE) | |
|  |  | Large Dependence Emphasis (LDE) | |
|  |  | Large Dependence High Gray Level Emphasis (LDHGLE) | |
|  |  | Large Dependence Low Gray Level Emphasis (LDLGLE) | |
|  |  | Low Gray Level Emphasis (LGLE) | |
|  |  | Small Dependence Emphasis (SDE) | |
|  |  | Small Dependence High Gray Level Emphasis (SDHGLE) | |
|  |  | Small Dependence Low Gray Level Emphasis (SDLGLE) | |

(GLCM: Gray Level Co-occurrence Matrix; GLRLM: Gray Level Co-occurrence Matrix; GLSZM: Gray Level Size Zone Matrix; GLDM: Gray Level Dependence Matrix.)

**S3: Methods of each step in the radiomics study pipeline.**

All the work related to radiomics models developing was completed through Feature Explorer Pro (FAEPro, V 0.3.4) in Python (v 3.6.0, <https://python.org>)[3]. Most algorithms in FAEPro were implemented with scikit-learn 0.19 ([https://scikit-learn.org](https://scikit-learn.org/)). The source code is openly available on GitHub (<https://github.com/salan668/FAE.git>). Alternative methods at every step of the modeling process are listed in Supplementary Table 2[2].

**Supplementary Table 2. Alternative methods at every step of the modeling process.**

| Modeling Steps |  | Methods |
| --- | --- | --- |
| Normalization |  | MinMax-Normalizer |
|  |  | Mean-Normalizer |
| Dimension Reduction |  | Pearson Correlation Coefficient (PCC) |
|  |  | Principle Component Analysis (PCA) |
| Feature Selection |  | Recursive Feature Elimination (RFE) |
|  |  | Analysis of Variance (ANOVA) |
|  |  | Kruskal-Wallis Test (KWT) |
|  |  | Relief |
| Classification |  | Least Absolute Shrinkage and Selection Operator (LASSO) |
|  |  | Random Forest (RF) |
|  |  | Support Vector Machine (SVM) |
|  |  | Decision Tree |
|  |  | ExtraTrees |
|  |  | Adaboost |
|  |  | Logistic Regression (LR)  GradientBoosting  LightGBM |
|  |  | CatBoost |

**S4: The parameter settings of each classifier**

1. **LRLasso (sklearn 0.24.1)**

penalty='l1',

Dual=False,

tol =0.01,

C=1.0,

fit_intercept=True,

intercept_scaling,

class_weight=None,

random_state=None,

solver ='liblinear',

max_iter=100,

multi_class='auto',

verbose=0,

warm_start=False,

n_jobs=None,

l1_ratio=None.

1. **RF (sklearn 0.24.1)**

n_estimators=200,

criterion="gini",

max_depth=None,

min_samples_split=2

min_samples_leaf=1,

min_weight_fraction_leaf=0.0,

max_features="auto",

max_leaf_nodes=None,

min_impurity_decrease=0.0,

min_impurity_split=None,

bootstrap=True,

oob_score=False,

n_jobs=None,

random_state=None,

verbose=0,

warm_start=False,

class_weight=None

ccp_alpha=0.0,

max_samples=None.

1. **SVM (sklearn 0.24.1)**

C=1.0,

Kernel='rbf',

Degree=3,

Gamma='scale',

coef0=0.0,

shrinking=True,

probability=True,

tol=1e-3,

cache_size=200,

class_weight=None,

verbose=False,

max_iter=-1,

decision_function_shape='ovr',

break_ties=False,

random_state.

1. **DecisionTree (sklearn 0.24.1)**

criterion="gini",

splitter="best",

max_depth=None,

min_samples_split=2,

min_samples_leaf=1,

min_weight_fraction_leaf=0.0,

max_features=None,

random_state=None,

max_leaf_nodes=None,

min_impurity_decrease=0.0,

min_impurity_split=0.0,

class_weight=None,

ccp_alpha=0.0.

1. **ExtraTrees (sklearn 0.24.1)**

n_estimators=100,

criterion="gini",

max_depth=None,

min_samples_split=2,

min_samples_leaf=1,

min_weight_fraction_leaf=0.0,

max_features ="auto",

max_leaf_nodes=None,

min_impurity_decrease=0.0,

min_impurity_split=None,

bootstrap=False,

oob_score=False,

n_jobs=None,

random_state=None,

verbose=0,

warm_start=False,

class_weight=None,

ccp_alpha=0.0,

max_samples=None.

1. **AdaBoost (sklearn 0.24.1)**

base_estimator=None,

n_estimators=50,

learning_rate=0.2,

algorithm='SAMME.R',

random_state=None.

1. **LogisticRegression (sklearn 0.24.1)**

penalty='none',

Dual=False,

tol =0.01,

C=1.0,

fit_intercept=True,

intercept_scaling,

class_weight=None,

random_state=None,

solver ='saga',

max_iter=100,

multi_class='auto',

verbose=0,

warm_start=False,

n_jobs=None,

l1_ratio=None.

1. **CatBoost (sklearn 0.24.1, catboost 0.25.1)**

iterations=None,

learning_rate=None,

depth=None,

l2_leaf_reg=None,

model_size_reg=None,

rsm=None,

loss_function=None,

border_count=None,

feature_border_type=None,

per_float_feature_quantization=None,

input_borders=None,

output_borders=None,

fold_permutation_block=None,

od_pval=None,

od_wait=None,

od_type=None,

nan_mode=None,

counter_calc_method=None,

leaf_estimation_iterations=None,

leaf_estimation_method=None,

thread_count=None,

random_seed=None,

use_best_model=None,

best_model_min_trees=None,

verbose=None,

silent=None,

logging_level=None,

metric_period=None,

ctr_leaf_count_limit=None,

store_all_simple_ctr=None,

max_ctr_complexity=None,

has_time=None,

allow_const_label=None,

target_border=None,

classes_count=None,

class_weights=None,

auto_class_weights=None,

class_names=None,

one_hot_max_size=None,

random_strength=None,

name=None,

ignored_features=None,

train_dir=None,

custom_loss=None,

custom_metric=None,

eval_metric=None,

bagging_temperature=None,

save_snapshot=None,

snapshot_file=None,

snapshot_interval=None,

fold_len_multiplier=None,

used_ram_limit=None,

gpu_ram_part=None,

pinned_memory_size=None,

allow_writing_files=None,

final_ctr_computation_mode=None,

approx_on_full_history=None,

boosting_type=None,

simple_ctr=None,

combinations_ctr=None,

per_feature_ctr=None,

ctr_description=None,

ctr_target_border_count=None,

task_type=None,

device_config=None,

devices=None,

bootstrap_type=None,

subsample=None,

mvs_reg=None,

sampling_unit=None,

sampling_frequency=None,

dev_score_calc_obj_block_size=None,

dev_efb_max_buckets=None,

sparse_features_conflict_fraction=None,

max_depth=None,

n_estimators=None,

num_boost_round=None,

num_trees=None,

colsample_bylevel=None,

random_state=None,

reg_lambda=None,

objective=None,

eta=None,

max_bin=None,

scale_pos_weight=None,

gpu_cat_features_storage=None,

data_partition=None,

metadata=None,

early_stopping_rounds=None,

cat_features=None,

grow_policy=None,

min_data_in_leaf=None,

min_child_samples=None,

max_leaves=None,

num_leaves=None,

score_function=None,

leaf_estimation_backtracking=None,

ctr_history_unit=None,

monotone_constraints=None,

feature_weights=None,

penalties_coefficient=None,

first_feature_use_penalties=None,

per_object_feature_penalties=None,

model_shrink_rate=None,

model_shrink_mode=None,

langevin=None,

diffusion_temperature=None,

posterior_sampling=None,

boost_from_average=None,

text_features=None,

tokenizers=None,

dictionaries=None,

feature_calcers=None,

text_processing=None,

embedding_features=None.

1. **LightGBM (sklearn 0.24.1, lightgbm 3.2.1)**

boosting_type='gbdt',

num_leaves=31,

max_depth=-1,

learning_rate=0.1,

n_estimators=100,

subsample_for_bin=200000,

objective=None,

class_weight=None,

min_split_gain =0,

min_child_weight=1e-3,

min_child_samples=20,

subsample=1,

subsample_freq=0,

colsample_bytree=1,

reg_alpha=0,

reg_lambda=0,

random_state=None,

n_jobs=-1,

importance_type='split'.

1. **GrandientBoosting (sklearn 0.24.1)**

loss ='deviance',

learning_rate=0.1,

n_estimators=100,

subsample=1.0,

criterion='friedman_mse',

min_samples_split=2,

min_samples_leaf=1,

min_weight_fraction_leaf=0.0,

max_depth=3,

min_impurity_decrease=0.0,

min_impurity_split =None,

init=None,

random_state=None,

max_features=None,

verbose=0,

max_leaf_nodes=None,

warm_start =False,

validation_fraction=0.1,

n_iter_no_change=None,

tol=1e-4,

ccp_alpha=0.0.

**S5: Details of the radiomics modeling pipelines**

*Normalization：Mean-Normalizer*

The function of Mean-Normalization is to reduce the fluctuation of the sample data so that the gradient descent can find a ‘shortcut’ more quickly to reach the global minimum. Make the sample data has also changed significantly in a smaller range. The function is$: x^{*}=\frac{x-\mu}{max-min}$ (μ is the mean of all sample data).

Dimension reduction: PCC

PCC is used to measure the degree of linear correlation between two features, if the PCC value of the two features was larger than 0.9, one of the features will be removed randomly. In this way, the dimension of the features is reduced and the features still retain the original classification.

Dimension reduction: PCA

PCA is a mathematical dimension reduction method that uses an orthogonal transformation to convert a series of linearly related variables into a set of new linearly unrelated variables. The meaning of these data is different from the original data, but contains most of the previous data, and has a lower dimension, which is convenient for further analysis.

Feature selection: RFE

Recursive feature elimination (RFE) is a common feature selection method. It works by removing features recursively and building a model on the remaining features. It uses the accuracy of the model to determine which features (or combinations of features) contribute more to the prediction results.

Feature selection: Relief

Relief is a feature weighting algorithm, which assigns different weights to features according to the correlation of each feature to a category, then features whose weight is less than a certain threshold will be removed.

Feature selection: ANOVA

Analysis of variance (ANOVA) is used to explore the significant features corresponding to the labels. The F-value was calculated to evaluate the relationship between the features and the label. The features were sorted by the corresponding F-value and a specific number of features were selected to build models.

Classifier: Adaboost

Adaboost is an iterative algorithm. Its core idea is to train different classifiers (weak classifiers) for the same training set, and then combine these weak classifiers to form a stronger final classifier (strong classifier).

Classifier: SVM

Support Vector Machine (SVM) is a generalized linear classifier that binary classification of data according to supervised learning. Its decision boundary is the maximum margin for solving the learning sample. Hyperplane. SVM uses the hinge loss function to calculate empirical risk and adds a regularization term to the solution system to optimize structural risk. It is a classifier with sparsity and robustness. SVM can perform non-linear classification through the kernel method, which is one of the common kernel learning methods.

**Supplementary Table 3. Key features used in the four radiomics models.**

| Model name | Key features |
| --- | --- |
| Model 1 | wavelet-HLH_glszm_LargeAreaHighGrayLevelEmphasis_^DCE__3.*.nii.gz |
|  | wavelet-LHH_glcm_MCC_^DCE__3.*.nii.gz |
|  | wavelet-HHH_glcm_ClusterShade_^DCE__3.*.nii.gz |
|  | Wavelet-HHL_glcm_ClusterProminence_^DCE__3.*.nii.gz |
|  | log-sigma-5-0-mm-3D_glcm_Imc1_^DCE__3.*.nii.gz |
|  | wavelet-HHL_glcm_ClusterShade_^DCE__3.*.nii.gz |
|  | log-sigma-5-0-mm-3D_firstorder_Kurtosis_^DCE__3.*.nii.gz |
|  | wavelet-HHH_gldm_SmallDependenceHighGrayLevelEmphasis_^DCE__3.*.nii.gz |
|  | wavelet-HLL_firstorder_Skewness_^DCE__3.*.nii.gz |
|  | wavelet-LLL_glcm_MCC_^DCE__3.*.nii.gz |
| Model 2 | PCA_feature_19 |
|  | PCA_feature_20 |
|  | PCA_feature_6 |
|  | PCA_feature_25 |
|  | PCA_feature_29 |
|  | PCA_feature_9 |
|  | PCA_feature_15 |
|  | PCA_feature_35 |
|  | PCA_feature_12 |
|  | PCA_feature_43 |
|  | PCA_feature_94 |
| Model 3 | PCA_feature_7 |
|  | PCA_feature_26 |
|  | PCA_feature_40 |
|  | PCA_feature_60 |
|  | PCA_feature_77 |
|  | PCA_feature_90 |
| Model 4 | PCA_feature_9 |
|  | PCA_feature_29 |
|  | PCA_feature_42 |
|  | PCA_feature_55 |
|  | PCA_feature_84 |
|  | PCA_feature_97 |

**References**

1. van Griethuysen JJM, Fedorov A, Parmar C, Hosny A, Aucoin N, Narayan V, Beets-Tan RGH, Fillion-Robin JC, Pieper S, Aerts H: **Computational Radiomics System to Decode the Radiographic Phenotype**. *Cancer Res* 2017, **77**(21):e104-e107.

2. Han C, Ma S, Liu X, Liu Y, Li C, Zhang Y, Zhang X, Wang X: **Radiomics Models Based on Apparent Diffusion Coefficient Maps for the Prediction of High-Grade Prostate Cancer at Radical Prostatectomy: Comparison With Preoperative Biopsy**. *Journal of magnetic resonance imaging : JMRI* 2021.

3. Song Y, Zhang J, Zhang YD, Hou Y, Yan X, Wang Y, Zhou M, Yao YF, Yang G: **FeAture Explorer (FAE): A tool for developing and comparing radiomics models**. *PLoS One* 2020, **15**(8):e0237587.
